# The Function of the MEF2 Family of Transcription Factors in Cardiac Development, Cardiogenomics, and Direct Reprogramming

**DOI:** 10.3390/jcdd3030026

**Published:** 2016-08-11

**Authors:** Cody A. Desjardins, Francisco J. Naya

**Affiliations:** Department of Biology, Program in Cell and Molecular Biology, Boston University, 24 Cummington Mall Boston, Boston, MA 02215, USA; cdesjard@bu.edu

**Keywords:** transcription factor, MEF2, cardiac muscle, development, genomics, reprogramming

## Abstract

Proper formation of the mammalian heart requires precise spatiotemporal transcriptional regulation of gene programs in cardiomyocytes. Sophisticated regulatory networks have evolved to not only integrate the activities of distinct transcription factors to control tissue-specific gene programs but also, in many instances, to incorporate multiple members within these transcription factor families to ensure accuracy and specificity in the system. Unsurprisingly, perturbations in this elaborate transcriptional circuitry can lead to severe cardiac abnormalities. Myocyte enhancer factor–2 (MEF2) transcription factor belongs to the evolutionarily conserved cardiac gene regulatory network. Given its central role in muscle gene regulation and its evolutionary conservation, MEF2 is considered one of only a few core cardiac transcription factors. In addition to its firmly established role as a differentiation factor, MEF2 regulates wide variety of, sometimes antagonistic, cellular processes such as cell survival and death. Vertebrate genomes encode multiple MEF2 family members thereby expanding the transcriptional potential of this core transcription factor in the heart. This review highlights the requirement of the MEF2 family and their orthologs in cardiac development in diverse animal model systems. Furthermore, we describe the recently characterized role of MEF2 in direct reprogramming and genome-wide cardiomyocyte gene regulation. A thorough understanding of the regulatory functions of the MEF2 family in cardiac development and cardiogenomics is required in order to develop effective therapeutic strategies to repair the diseased heart.

## 1. Introduction

Development of the mammalian heart is a complex process that requires precise coordination of cardiomyocyte specification, proliferation, differentiation, and growth. Additionally, the heart consists of multiple muscle and non-muscle cell types, derived from distinct precursor populations, which must assemble at the right place and time to form the specialized, anatomical structures, i.e., chambers, valves, blood vessels, of this vital organ. In order to accomplish this enormous task the cardiac gene regulatory network must interconnect diverse signaling pathways and their downstream effectors to ensure proper spatiotemporal gene expression. Not surprisingly, perturbations in this developmental program result in cardiac defects that are associated with abnormal gene expression patterns. A number of congenital cardiac abnormalities have been linked to mutations in transcription factors that affect their ability to properly regulate gene expression [1,2]. Moreover, many transcription factors important for cardiac development have been shown to function in pathological gene reprogramming in the diseased heart [3]. Thus, a comprehensive understanding of transcription factor function in the heart will shed light on their broad gene regulatory potential and how this activity can be harnessed to promote cardiomyocyte survival, proliferation, or differentiation.

MEF2 is a transcription factor family that plays a prominent role in cardiovascular development and differentiation [4,5,6]. It is also one of a small cohort of core transcription factors that form an evolutionarily conserved gene regulatory framework for cardiac development [7]. Whereas invertebrate genomes such as flies and worms encode a single MEF2, vertebrate MEF2 is encoded by four genes, *Mef2a*, *-b*, *-c*, and *-d*, that are coexpressed in cardiac muscle. The vertebrate MEF2 proteins display extensive sequence similarity in their DNA binding domains but diverge substantially in their transactivation domains. Moreover, a number of transcript variants can be generated by alternative splicing [8], presumably leading to MEF2 protein isoforms with distinct transcriptional activities. Their ability to form homo- and heterodimers also adds an important layer of specificity to their transcriptional regulatory function. Expansion of MEF2 transcriptional potential and target gene specificity through alternative isoforms and unique dimeric combinations has likely contributed to the increased physiological and structural complexity of the heart over evolutionary time.

Dissection of the specific roles of MEF2 isoforms in cardiac gene regulation and function is complicated by functional redundancy within the family and the inability to distinguish homo- or heterodimeric combinations on target genes. Additionally, in most instances in vitro reporter assays fail to recapitulate in vivo regulatory differences between the MEF2 family members. Despite these obstacles, analysis of gain-of- and loss-of-function mutations in model systems have revealed dramatically different cardiac phenotypes and dysregulated gene expression profiles for the vertebrate MEF2 isoforms suggesting that family members harbor unique regulatory activities.

This review highlights the varied phenotypes of MEF2 misexpression in the heart of model systems and recent knowledge we have acquired about the genome-wide role of the MEF2 family of transcription factors in cardiomyocyte gene regulation. While MEF2 has been the subject of a number of comprehensive and excellent reviews on the evolution of the heart, organ development, post-transcriptional regulation, pathological remodeling in the heart, cancer, and neurogenesis, this detailed review focuses on the developmental, postnatal, and genomic role of this core transcription factor and its orthologs in the heart. Expansion of the MEF2 family in vertebrates has likely allowed individual members to evolve distinct roles in muscle gene regulation. Given the pervasive role of the MEF2 family in the cardiovascular system, this review will pay particular attention to the distinct processes regulated by MEF2 in the heart. We begin by describing the requirement of MEF2 in cardiac development in invertebrate and vertebrate animals. We then describe how recent genome scale approaches have revealed the nodal role of the MEF2 family of transcription factors in cardiomyocyte gene regulatory networks. Additionally, we review a growing body of work focusing on the role of the MEF2 family in cardiomyocyte transdifferentiation, i.e., direct reprogramming. A deeper appreciation of the cardiac phenotypes associated with mutations in MEF2 orthologs in diverse model systems and the gene programs regulated by this family of transcription factors will be essential for the development of therapeutic approaches to treat heart disease.

## 2. Cardiac Development and Differentiation—Model Organisms

### 2.1. Invertebrates

#### 2.1.1. Flies (*D. melanogaster*)

This classic and powerful animal model system has been extensively used to decipher the gene regulatory networks of cardiac development and function [9,10]. Drosophila has an open circulatory system and hemolymph is pumped throughout the organism by the dorsal vessel, a linear heart-like tube. Despite its apparent simplicity, the dorsal vessel has a well-defined aorta and primary contractile region, and consists of two cardiac cell types: cardioblasts and non-muscle pericardial cells. Drosophila *Mef2* (*D-Mef2*) is expressed in cardioblasts, the lineage that will give rise to the myocardial cells that perform the contractile activity of the dorsal vessel [11].

##### Function

Seminal studies in this invertebrate animal model system unambiguously demonstrated the importance of MEF2 for the differentiation of all muscle types: cardiac, somatic, and visceral. Null mutations of *D-Mef2* were generated by genetic deletion or chemical mutagenesis with similar phenotypic results [12,13]. Although the dorsal vessel formed in both mutant lines of flies, D-Mef2 mutant cardiomyocytes failed to express differentiation genes [12,13]. Recent studies have now implicated D-Mef2 in the regulation of cardiac cell fate. D-Mef2 was shown to collaborate with the cardiac transcription factors Tinman (Nkx2.5) and Pannier (GATA4) to expand the cardiogenic pool of cells from mesoderm [14].

#### 2.1.2. Nematodes (*C. elegans*)

Nematodes do not have a heart but striated muscle cells in the pharynx exhibit cardiomyocyte-like contractile properties. Like flies, nematodes encode a single *Mef2* gene that is ubiquitously expressed [15]. Interestingly, two different mutant alleles of *Mef2* were generated but these mutations did not overtly impair embryonic development or muscle differentiation [15]. Epistatic analysis was performed to determine the extent to which Mef2 could modulate known muscle phenotypes. Genetic crosses of *CeMef2* mutants to *MyoD*, *Twist*, or *pha-1* mutant worms defective in mesoderm or muscle development failed to exacerbate those muscle defects. Despite the lack of obvious morphological defects a more detailed genomic and cellular analysis may be needed to address its specific requirement in muscle in this organism.

#### 2.1.3. Ascidians (*C. intestinalis*)

The sea squirt *Ciona intestinalis* is an emerging chordate model system used to dissect the gene regulatory networks in cardiac morphogenesis given the simplicity of its genome and presence of a cardiac-like tubular structure. Current investigations suggest that the cardiogenic programs are conserved between *Ciona* and vertebrates [16]. Although there is no information regarding the cardiac function of Mef2 in this model system, a large scale expression analysis of candidate transcription factors included Mef2 as a representative muscle transcription factor. Comprehensive in situ hybridization analysis found that *Mef2* transcripts were first detected in the epidermis of the late gastrula stage embryos [17]. *Mef2* transcripts were subsequently detected at the early tailbud stage in the nervous system and endoderm, and in muscle at the late tailbud stage. Given the tools that have been developed for *Ciona* in recent years, this animal model is well-positioned to address the evolutionarily conserved role of Mef2 in cardiac gene regulation and development.

### 2.2. Vertebrates

#### 2.2.1. Zebrafish (*D. rerio*)

Zebrafish have a two chambered heart, a single atrium and ventricle [18]. The zebrafish genome encodes orthologs of the four mammalian *Mef2* genes, and their expression is largely restricted to muscle lineages and the brain [19,20,21]. Zebrafish also encode *mef2a* and *mef2c* paralogs perhaps due to additional genome duplication events in this evolutionary branch. The paralogs *mef2ca* and *mef2cb* are most closely related to *Mef2c* whereas *mef2aa* and *mef2ab* are likely orthologs of *Mef2a* [22]. In situ expression analysis revealed that *mef2c* (*mef2ca*) transcripts are the earliest detectable *mef2* transcripts expressed in the cardiac primordia [16 h post fertilization (hpf)] whereas *mef2a* (*mef2aa*) transcripts are detected a few hours later (18 hpf) at the time of myocardial differentiation. *Mef2a* and *mef2c* expression is sustained through late development (48 hpf). Surprisingly, despite its abundant expression in skeletal muscle [21], *mef2d* transcripts have not been detected at early cardiac developmental time points. Expression analysis of the apparent *Mef2b* homolog remains to be determined.

##### Function

Several groups have analyzed the in vivo role of zebrafish *mef2* genes using morpholino oligonucleotides and/or mutant lines derived from large scale mutagenesis screens. Mef2a morphants were generated by using two different morpholino oligonucleotides. These morphants display normal cardiac morphology but Mef2a-deficiency caused a significant reduction in cardiac contractility and disorganized sarcomere structure [23]. In addition, expression of *mef2a* was found to be downregulated in Bmp2 morphants, mutant zebrafish that also display cardiac contractility defects and myofibrillar disorganization [24]. Curiously, while Mef2a overexpression did not rescue the Bmp2 morphant phenotype it was able to rescue cardiac contractility defects in embryos treated with Noggin, a BMP inhibitor [24]. These results suggest that a Bmp2-Mef2a molecular pathway is required for proper cardiomyocyte differentiation and/or function. Indeed, Mef2a expression has been shown to be regulated by BMP-2 upon differentiation of the cardiomyocyte-like CL6 cell line, a subclone derived from P19 embryonal carcinoma cells [25]. In the future it would be interesting to know whether MEF2A functions downstream of the TGFβ/BMP signaling pathway in mammalian cardiac morphogenesis.

The zebrafish *mef2c* genes have also been extensively analyzed in cardiac development. Morpholino inhibition of the *mef2cb* paralog resulted in defects in the second (anterior) heart field as these morphants lacked a subset of cardiomyocytes in the arterial pole of the developing heart [20]. In a subsequent study, the role of both *mef2c* paralogs, *mef2ca* and *mef2cb*, were analyzed using gain-of-function and loss-of-function approaches. A genetic mutation in *mef2ca* (b1086) resulted in delayed cardiomyocyte differentiation marker expression, but ultimately the hearts developed normally [22]. By contrast, *mef2cb* morphants displayed shortened hearts characterized by reduced atrial and ventricular volume. However, a *mef2cb* mutant allele (fh288), which causes a premature stop in the MADS box DNA binding domain, displayed no overt cardiac phenotype. Combinatorial *mef2ca* and *mef2cb* deficiency, using various combinations of individual *mef2c* genomic mutant lines and morpholino approaches, resulted in pericardial edema, and impaired cardiomyocyte differentiation and heart tube formation in a substantial percentage of mutant embryos [22]. A broader ablation of all *mef2* isoforms using a single morpholino (*mef2d/c*) yielded a more severe cardiac phenotype [22]. These morphants lacked all myosin heavy chain expression, and most cardiomyocytes failed to differentiate. As the vast majority of *mef2* mutant analyses in this animal model were performed with morpholino oligonucleotides, the distinct cardiac phenotypes associated with each isoform knockdown will need to be further clarified using mutagenized zebrafish lines harboring bona fide loss-of-function or null alleles of the *mef2* genes.

Conversely, to determine whether overexpression of Mef2c is sufficient to promote cardiomyocyte determination the Mef2cb isoform was injected at the 1–2 cell stage of zebrafish development. Curiously, Mef2cb caused ectopic skeletal, but not cardiac, muscle formation in the head region, suggesting that we still fail to have a complete molecular understanding of the cardiac regulatory role of Mef2 in zebrafish.

#### 2.2.2. Frogs (*X. laevis*)

Amphibians have a three chambered heart, with two atria and one ventricle [18]. *Mef2* orthologs for *mef2a*, *-c*, and *-d* have been identified in the *Xenopus* genome and their temporal expression patterns have been analyzed. Despite abundant expression of *mef2a* and *mef2d* transcripts in the adult heart, prior investigations failed to detect expression of these genes in the embryonic heart [26]. Recently, however, expression of *mef2d* but not *mef2a* was clearly detected in the presumptive heart region of neurula embryos [27]. Additionally, *Xenopus* cardiac fate mapping has detected *mef2c* and *mef2d* transcripts in cardiac progenitor populations [28,29]. The lack of expressed sequence tags (ESTs) and the absence of *mef2b* genomic regions, as determined by synteny analysis with mouse and human genomes, suggests that *mef2b* does not exist in the frog genome [29].

##### Function

The roles of *mef2* isoforms have been investigated in cardiac development in frog embryos. Microinjection of *mef2a* mRNA in two-cell stage Xenopus embryos resulted in precocious expression of sarcomeric αMHC gene at stage 14, which later resulted in an enlarged heart phenotype [30]. These results suggest that Mef2a has cardiogenic inducing activity in frogs. Morpholino-mediated inhibition of either *mef2c* or *mef2d* caused defects in cardiac looping morphogenesis, chamber expansion, and dysregulation in cardiac marker gene expression [29]. Interestingly, rescue experiments revealed that Mef2d overexpression compensated for Mef2c-deficiency, but Mef2c overexpression was unable to rescue the Mef2d phenotype [29]. Although the Mef2 factors have not been extensively characterized in this model system the above results suggest isoform-specific functions in cardiogenesis. A summary of MEF2 mutant phenotypes in models systems with one, two, and three-chambered hearts are listed in Table 1.

#### 2.2.3. Avian (*Gallus gallus*)

Birds, like mammals, have a four chambered heart consisting of two atrial and two ventricular chambers [18]. Recently, a detailed temporal expression analysis of the four *Mef2* genes was performed in developing chick embryos [31]. Unlike frogs and rodents, *Mef2a* is the first *Mef2* gene to be expressed (in the primitive streak) and is subsequently coexpressed with *Mef2c* and *Mef2d* in the precardiac mesoderm of HH stage 7–8 embryos, and continues through the primitive heart in HH stage 10 [31,32]. *Mef2b* is highly expressed in cardiac precursors and primitive heart tube, and by HH stage 14 all four *Mef2* factors are coexpressed in all four cardiac chambers. Interestingly, *Mef2c* expression has been reported in the Purkinje fibers of the heart, specialized cardiomyocytes with conduction properties [33]. Functional studies investigating the specific requirement of *Mef2* genes in avian cardiac development have not been performed.

### 2.3. Rodents—Mice (M. musculus), Rat (R. norvegicus)

In mammalian embryos the heart begins as a curved stripe of cardiac precursor cells, i.e., the cardiac crescent, that have been specified to develop into this vital organ. Spatiotemporal in situ hybridization analysis on developing mouse embryos revealed that *Mef2b* and *Mef2c* are the first *Mef2* isoforms expressed in the cardiac mesoderm at embryonic day 7.5 (E7.5) prior to expression of sarcomeric genes such as cardiac α-actin [34,35]. *Mef2c* is also expressed in the sinus venosus which contributes to the cardiac atria. *Mef2a* and *Mef2d* are subsequently expressed in the linear heart tube between E8.0 and E8.5, and after E8.5 all four *Mef2* genes are expressed throughout the developing heart. *Mef2b* and *Mef2c* expression begins to decline at E11.5, whereas *Mef2a* and *Mef2d* continue to be expressed. Additionally, *Mef2a* and *Mef2c* are expressed in the cardiac outflow tract as early as E9.5 and their expression seems far more prominent than that of *Mef2d* [34]. Postnatally, *Mef2a* and *Mef2d* are the most abundant isoforms in the heart. However, *Mef2c* expression has also been detected in postnatal cardiomyocytes in homeostasis [36].

The spatiotemporal readout of MEF2 transcriptional activity in developing and adult mice was determined by generating transgenic lines harboring the *lacZ* gene driven by 3 copies of the *desmin* MEF2 site and surrounding flanking sequences [37]. Presumably all MEF2 isoforms and dimeric combinations activate the reporter in vivo but this has never been formally tested. Reporter expression was restricted primarily to the muscle and neuronal lineages, tissues expressing high levels of MEF2. In the heart, activity of the transgene was detected in a cluster of cardiogenic precursors in the cardiac crescent as early as E7.5 consistent with the expression of *Mef2c*. At E8.5, βgal expression appeared to be restricted to myocytes and was observed in the aortic sac, conotruncus, and the developing ventricles and atria. Transgene expression continued to be detected uniformly throughout the developing heart in both the atrial and ventricular chambers through E14.5. However, despite abundant expression of *Mef2a* and *Mef2d* at this timepoint and postnatally, βgal expression began to decline in late fetal cardiac development, and was greatly diminished postnatally. The reduction in postnatal MEF2-dependent reporter expression stems, in part, from the repression of MEF2 activity by histone deactylases (HDACs) [6,38]. Given the important role of this pathway in the heart, the modulation of MEF2 activity by HDACs is discussed following the description of mammalian MEF2 isoform function. Despite the potent repression of MEF2 activity by HDACs, high expression levels of MEF2A and MEF2D and robust in vitro DNA binding activity in postnatal cardiac extracts, suggest that MEF2 protein isoforms still retain a basal and essential transcriptional function in muscle gene regulation in postnatal cardiomyocyte homeostasis. Indeed, as described below, MEF2A and MEF2D are required for proper gene regulation and survival in neonatal cardiomyocytes.

#### 2.3.1. MEF2A

To determine the in vivo role of the mammalian MEF2 transcription factor family knockout (KO) mice have been generated for three of the four murine MEF2 genes by deleting the second coding exon (encoding the MADS box DNA binding and MEF2 domains). This deletion effectively renders these mutations as loss-of-function alleles. Global deletion of MEF2A showed an important role for this isoform in the postnatal heart. The majority of MEF2A mutant mice die suddenly in the perinatal period with cardiac enlargement [39]. Detailed analyses of cardiomyocytes from these mutants revealed severe myofibrillar defects likely resulting from the misregulation of a MEF2A-dependent costamere gene program [40,41,42]. Neonatal cardiomyocytes depleted of MEF2A using RNA interference (RNAi) also display widespread apoptosis probably resulting from deficiencies in focal adhesion contacts [42]. To understand the transcriptional mechanisms involved in MEF2A-depedent regulation of costamere genes our group used bioinformatics to identify candidate cofactor binding sites in MEF2A-regulated costamere genes. One of these sites belonged to the Egr1 transcription factor, a factor known to play a regulatory role in cardiovascular pathology [43]. Functional characterization of this computational prediction revealed that Egr1 and MEF2A interact and represses MEF2-dependent reporters [44]. Reinforcing these observations, overexpression and depletion of Egr1 in neonatal myocytes resulted in downregulated and upregulated costamere gene expression, respectively. Finally, while a small percentage of MEF2A KO mice are viable and survive to adulthood, their hearts display mitochondrial deficiency and conduction defects. The genetic basis of survival in a subset of MEF2A KO mice and molecular mechanisms of the mitochondrial and conduction abnormalities remain to be determined.

Interestingly, dysregulated expression of MEF2A has been reported in the embryonic heart of a cardiac-specific knockout of focal adhesion kinase, FAK [45]. Floxed FAK mice were crossed to MLC2a-Cre and this resulted in lethality during late fetal development. Mutant mice at E14.5 displayed thin ventricular walls, disorganized myofibrils, and decreased cardiomyocyte proliferation. At the molecular level this defect was associated with significant downregulation of MEF2A. Conversely, overexpression of FAK in NIH 3T3 cells induced significant upregulation of MEF2A [45]. Consistent with the above observations, antisense oligonucleotide inhibition of FAK attenuated mechanical stress/stretch-induced MEF2 DNA binding activity in neonatal cardiomyocytes [46]. Detailed structural analysis of this pathway in mechanically stressed myocytes has now revealed a direct association between FAK and MEF2 [47]. Taken together, these results point to an essential role for MEF2A in a focal adhesion/costamere regulatory circuit in cardiomyocyte differentiation and stress.

#### 2.3.2. MEF2C

Of the mammalian MEF2 genes, the MEF2C knockout is the only one that results in embryonic lethality. MEF2C knockout mice display defective cardiac looping morphogenesis and vascular malformations [48,49,50]. However, cardiomyocyte-specific deletion of MEF2C at around E10.5 using αMHC-Cre transgenic mice results in viability and normal cardiac development [51]. These findings suggest that this isoform is dispensable after early embryonic development or that its transcriptional function is compensated for by the remaining MEF2 factors. Given the similarities in their embryonic cardiac developmental defects, mice with a combinatorial deficiency of MEF2C and the cardiac transcription factor Nkx2.5 were generated. These double homozygous null embryos developed a heart with a single cardiac chamber expressing atrial and second heart field markers but lacking ventricular markers [52]. These results indicate that MEF2C and Nkx2.5 function in the same genetic pathway for the specification and differentiation of ventricular myocytes. Recently, floxed MEF2C have been used to delete this mammalian isoform in the anterior second heart field. These mice display a wide range of outflow tract defects such as overriding aorta and double outlet right ventricle reinforcing the important role of this mammalian MEF2 isoform in embryonic cardiac morphogenesis [53].

Given the early and essential role of MEF2C in murine cardiac development, the mechanisms by which its expression is regulated in vivo has received considerable attention and led to substantial insight into the genetic circuitry of cardiac morphogenesis. These comprehensive gene regulatory analyses have resulted in the identification of novel enhancers and upstream effectors that regulate MEF2C expression in the cardiovascular system, and enabled investigators to acquire a more refined understanding of its placement in the hierarchy of cardiac transcriptional networks [54].

Like other vertebrate animals the mammalian heart is derived from two distinct mesodermal regions in mammals known as the first and second (anterior) heart fields [55]. Reporter analysis of several hundred kilobases of genomic sequence surrounding the murine MEF2C gene in transient transgenic mouse embryos has led to the discovery of several cardiovascular and other tissue-specific enhancers [56,57,58]. The activity of one of the cardiovascular enhancers was restricted to the anterior heart field (AHF), a region which harbors late differentiating cardiomyocyte precursors and gives rise to the outflow tract (OFT) and right ventricle (RV) [57]. This enhancer was shown to be regulated by both the Isl1 and GATA4, cardiac transcription factors that function in lineage specification and differentiation, respectively. Subsequently, another cardiovascular enhancer was shown to be expressed in the AHF and restricted to the OFT and RV regions of the developing mouse heart [59]. This intronic enhancer is TGFβ-responsive and cooperatively regulated by the Foxh1 and Nkx2.5 transcription factors [59]. In addition to dissecting the in vivo regulation of MEF2C expression, the *Mef2c*-AHF enhancer has become a valuable reagent used to drive the cardiac-specific and regional expression of Cre recombinase for the conditional deletion of numerous regulatory genes in the developing heart [60]. To date, although one study has analyzed the human MEF2A promoter in vitro [61], the MEF2C gene is the only mammalian MEF2 isoform for which we have a well-defined mechanistic understanding of its transcriptional regulation in vivo and integration in the gene regulatory network of the cardiovascular system.

#### 2.3.3. MEF2D

Global deletion of MEF2D was generated by crossing floxed MEF2D mice to *Meox*-Cre, which is active in the three germ layers. Loss-of-function of MEF2D resulted in viable mice with normal cardiac structure and function. However, when adult MEF2D KO mice were subjected to cardiac stressors these hearts display attenuated hypertrophy and fibrosis [62]. Additionally, despite the apparent lack of a cardiac phenotype in MEF2D-deficient hearts in homeostasis, we have shown that acute depletion of MEF2D in neonatal cardiomyocytes in vitro results in cell cycle re-entry and programmed cell death [63]. Perhaps these differences relate to the temporal requirement of MEF2D in postnatal myocytes or differences between in vitro and in vivo approaches in analyzing MEF2D deficiency.

**Figure 1:**
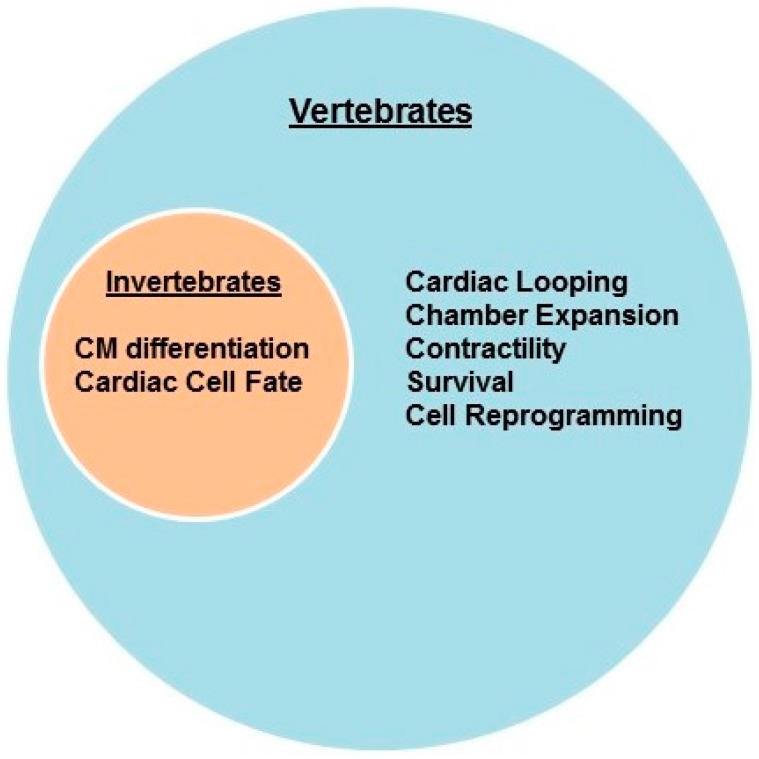
Schematic of process regulated by the MEF2 family of transcription factors. The core cardiac transcription factor MEF2 is a key regulator of cardiac development and some of the gene programs it regulates have been evolutionarily conserved. Invertebrate MEF2 and certain isoforms of vertebrate MEF2 have been shown to be important in regulating cytoarchitectural gene transcription (overlapping region). Non-overlapping regions depict those gene programs/cellular processes that have been shown to be regulated by either the invertebrate or vertebrate only MEF2 proteins.

The regulatory roles of alternatively spliced MEF2D isoforms have recently been investigated in muscle differentiation [64]. Two alternatively spliced MEF2D isoforms, α1 and α2, are expressed in skeletal muscle but the MEF2D α2 isoform appears to be required for differentiation. This α2 isoform includes a coding exon that is resistant to inhibitory phosphorylation mediated by protein kinase A (PKA) [65] thereby activating muscle transcription. Along these lines, a number of mammalian MEF2 splice isoforms have been previously described [66]. In particular, an exon (the β+ isoform) near the 3′ end of MEF2A, -C, and -D, resulted in more potent transactivation compared to MEF2 isoforms lacking this domain. Although the above experiments were performed in skeletal muscle, given the overlapping gene programs in striated muscle, it is tempting to speculate that alternatively spliced isoforms of MEF2 proteins display distinct regulatory properties in cardiomyocytes. Much remains to be determined regarding alternative splice isoforms within each of the MEF2 genes and their specific role in cardiac gene regulation. Comparison of MEF2 dependent cellular processes in the heart of invertebrate and vertebrate models systems is depicted in Figure 1.

#### 2.3.4. Additional Functional Studies Relating to MEF2 Misexpression and Activity

In the years following the reported links between MEF2 and chromatin modifiers [67,68,69], the MEF2-HDAC interaction has been the most extensively investigated pathway in pathophysiological processes in the heart. It is now firmly established that dissociation of class II HDACs from MEF2 downstream of hypertrophic signaling is a key event in the stimulation of postnatal MEF2 transcriptional activity [70,71,72], a process that ultimately contributes to the genomic reprogramming observed in cardiac pathology. Briefly, these pathological signals activate a number of protein kinases to promote sequestration of class II HDACs in the cytoplasm. Most, if not all, of these protein kinases such as calcium-calmodulin kinases and protein kinase D phosphorylate class II HDACs on specific amino acid residues which serve as docking sites for adaptor protein 14-3-3, resulting in their nuclear export and subsequent MEF2 activation [73,74]. Additionally, the PKA signaling pathway including the A-kinase anchoring protein, AKAP-Lbc, have been shown to modulate HDAC and MEF2 activity [75,76]. Apart from the pathological findings, the MEF2-HDAC pathway has been implicated in cardiovascular development. Inhibition or activation of HDACs in P19 cells was shown to enhance and impair cardiomyocyte differentiation, respectively, in part through the regulation of *Mef2c* expression [77]. In mice, global knockout of the G protein-coupled receptor APJ resulted in a spectrum of embryonic (E10.5–E12.5) cardiovascular defects and endocardial cells displayed increased HDAC4 and 5 nuclear localization [78]. Consistent with these results, overexpression of APJ and its ligand, apelin, in endothelial cells in vitro robustly induced MEF2 transcriptional activity.

To understand the collective requirement of MEF2 isoforms in cardiomyocytes, its transcriptional activity has been inhibited using dominant negative approaches but with different outcomes. Inhibition of MEF2 activity in cardiomyocytes derived from P19 embryonal carcinoma cells resulted in impaired differentiation [79]. This study generated a dominant negative form of MEF2 by fusing the DNA binding domain of MEF2C (amino acids 1–105) with the potent repressor domain from the Engrailed transcription factor (EnR), and regulated by the Nkx2.5 enhancer, which is active at the onset of cardiac development at E7.5. This impaired cardiomyocyte differentiation was supported in vivo by transient overexpression in mouse embryos which revealed two distinct phenotypes based on the severity of cardiac development [79]. The most severe transgenic embryos failed to form a heart but myosin heavy chain (MyHC) positive cardiomyocytes were detected. The less severe displayed thin walled myocardium. These results are consistent with an earlier study demonstrating the differentiation promoting effects of MEF2C when overexpressed in P19 embryonal carcinoma cells [80].

In contrast to the severe cardiac defects in transient mouse embryos, stable transgenic mice expressing dominant-negative forms of MEF2C did not interfere with cardiac development but instead a phenotype was observed postnatally. One study described attenuated post-natal growth of the myocardium overexpressing the DNA binding domain of MEF2C (amino acids 1–95) driven by the αMHC promoter [81]. In another independent study, stable transgenic mice harboring a different dominant negative MEF2 (MEF2C amino acids 1–117; DNA binding defective R24L mutant) had no measurable phenotypic effects on heart size or function [82]. However, there were key differences in the approaches that could account for the distinct phenotypes. The truncated MEF2C and MEF2C-EnR constructs are capable of DNA binding whereas the R24L mutant cannot bind to DNA. Moreover, MEF2C R24L transgene was induced using a “floxed ON” approach by crossing to αMHC-Cre transgenic mice. Despite its effectiveness in inducing loxP recombination in the embryonic heart transgene levels may not have been expressed at sufficiently high levels to induce a phenotype.

Individual mammalian MEF2 proteins have also been overexpressed in neonatal cardiomyocytes and in the heart. Overexpression of MEF2A, -C, or -D in neonatal cardiomyocytes in vitro induced similar morphological phenotypes characterized by sarcomere disorganization and focal elongation [83]. Overexpression of these MEF2 proteins individually in the mouse heart using the cardiomyocyte-specific αMHC promoter, which is active in development but massively upregulated post-natally, also resulted in comparable phenotypes. MEF2A and MEF2C overexpression induced dilated cardiomyopathy [40,82,83], whereas MEF2D overexpression resulted in atrial enlargement and extensive fibrosis [62]. While these results suggest functionally redundant roles of MEF2 family members in post-natal cardiomyocytes it should be noted that the proteins were expressed at supra-physiological levels potentially overcoming isoform-specific gene regulatory effects. A summary of MEF2 mutant phenotypes in mammalian models systems are listed in Table 2.

## 3. Functional Genomic Analysis of MEF2 in Striated Muscle

While there is no doubt that MEF2 is an essential transcription factor in the regulation of the cytoarchitectural gene program in muscle in virtually all animal model systems [4,5,84], a much broader gene regulatory function for MEF2 has been uncovered in recent years through comprehensive genome-wide studies. Initial studies were carried out primarily in *Drosophila* somatic muscle and the murine C2C12 skeletal myoblast cell line. Findings in these model systems expanded the traditional view of MEF2 from that of an exclusive transcriptional regulator of structural genes to one harboring broader regulatory function through its regulation of multiple gene programs in muscle. More recently, next generation genomic technologies have been applied to investigate the role of MEF2 in gene regulation, i.e., genome-wide binding and target gene expression, in cardiomyocytes on a global scale. Given the limitation in isoform-specific reagents for mammalian MEF2 proteins, the majority of the genomic studies performed in vertebrate model systems have focused primarily on a single MEF2 isoform or generalized its genome-wide role based on a representative member of the family.

The first in vivo, global analysis of MEF2 genome binding was performed in *Drosophila* [85]. This seminal study performed chromatin immunoprecipitation followed by genomic DNA microarray (ChIP-on chip) at multiple timepoints in muscle development. These experiments revealed hundreds of genomic regions bound by MEF2 at all stages of muscle development and not restricted to terminal differentiation. A number of these bound regions were found to function as enhancers in vivo and to drive MEF2-dependent reporter expression in somatic muscle. MEF2 bound to genomic regions predicted to regulate a variety of pathways in addition to structural genes including myoblast fusion, extracellular matrix-muscle attachment, and somatic and cardiac muscle identity genes such as those belonging to the Notch-Delta signaling pathway. Several MEF2 bound enhancers in the aforementioned study were previously identified in *Drosophila* by combining computational prediction of muscle enhancer elements with ChIP using a MEF2 antibody [86].

In vertebrates, genome-wide analyses on muscle regulatory factors including MEF2 were first performed in C2C12 skeletal myoblasts [87,88]. Consistent with the findings in *Drosophila*, both of these ChIP-on-chip studies revealed MEF2 binding was not limited to structural genes but included genes belonging to signal transduction cascades, transcription factors, muscle differentiation, and the neuromuscular junction. It is worth noting that while both groups used the same MEF2 antibody one was reported to be MEF2A-specific and the other MEF2C. Nevertheless, both studies arrive at the conclusion that the MEF2 transcription factor family regulates an array of cellular processes in muscle. As described earlier in this review, the distinct functions of MEF2D isoforms were examined in C2C12 myoblasts by subjecting MEF2D isoform specific antibodies to ChIP followed by high-throughput sequencing (ChIP-seq) [64]. Despite the different activities of each MEF2D alternatively spliced isoform in myogenic differentiation, this study revealed similar binding site preferences and extensive overlap of target genes bound by MEF2D isoforms suggesting additional regulatory mechanisms beyond DNA binding. Finally, our group performed a global analysis of the target genes regulated by the four mammalian MEF2 factors in C2C12 myoblasts [89]. In stark contrast to the longstanding notion of functional redundancy in the family, we discovered that only a small percentage of genes were coregulated by all four isoforms. Moreover, transcription factor binding site enrichment analysis of the promoters of these genes revealed that individual MEF2-regulated gene sets harbor a distinct cohort of coregulators. Because skeletal and cardiac muscle have overlapping structural and metabolic properties, it is a distinct possibility that cardiomyocyte gene programs are also differentially regulated by MEF2 family members.

To investigate genome-wide binding of the mammalian core cardiac transcription factors in cardiomyocytes, an integrative systems level analysis was performed in HL-1 cells, a cardiac atrial cell line. Using a variety of genome scale technologies such as ChIP-on-chip and RNAi, common and unique target genes regulated by GATA4, MEF2A, Nkx2.5, and SRF were determined [90]. This global analysis revealed that a different cohort of target genes in the myocyte genome were regulated by a distinct combination of cardiac transcription factors. For example, MEF2A and Nkx2.5 were found to coregulate genes belonging to muscle cell differentiation and heart looping pathways. A similar study used HL-1 cells to identify genomic binding of core cardiac transcription factors by inducible overexpression of biotinylation peptide-tagged versions of these regulatory factors, allowing biotinylation by BirA, followed by streptavidin pulldown and ChIP-seq [91]. These findings revealed co-occupancy of cardiac enhancers with various combinations of GATA4, MEF2A, TBX5, SRF, and Nkx2.5. Additionally, a number of cardiac enhancers associated with heart development and function were found to be coregulated by GATA4 and MEF2A. Given the important and common role of GATA and MEF2 transcription factor families in cardiac development and disease, further genomic dissection of this coregulatory pathway is warranted. Finally, genome-wide analysis of Tbx20 bound regions in the heart of transgenic mice overexpressing epitope-tagged Tbx20 revealed a significant enrichment of MEF2 binding sites in these enhancers [92]. Gene ontology analysis of these regions revealed genes associated with ion transport, calcium signaling, and contraction, cellular processes previously attributed to the MEF2 family.

Recently, ChIP-exo, a modification of ChIP-seq that improves sequencing resolution by digesting unbound DNA regions using exonuclease, was used to identify and compare the genome-wide, direct target genes of MEF2A in neonatal cardiomyocytes and C2C12 myoblasts [93]. While there was modest overlap in target genes, the MEF2A bound regions in cardiomyocytes had an overrepresentation of AP-1, CREB, BACH, and ERE transcription factor binding sites. Furthermore, gene ontology analysis revealed that the most significantly enriched genes bound by MEF2A in neonatal cardiomyocytes, are involved in actin organization. These results are consistent with the role of MEF2A the regulation of the costamere/focal adhesion complexes which are intimately associated with the actin cytoskeleton [42,84].

To understand the gene reprogramming that occurs in heart disease the epigenetic signature in pathological hypertrophy was evaluated by ChIP-seq. Pathological cardiac hypertrophy was induced by trans-aortic constriction (TAC), i.e., pressure overload. Cardiomyocytes were then isolated from these stressed hearts and subjected to ChIP using a panel of antibodies which recognize specific histone modifications [94]. The genomic pattern of modified histone H3 amino acid residues (acetylation, methylation) was analyzed to determine the potential redistribution of these epigenetic marks under disease-specific conditions. Enhancers activated in hypertrophy were determined and subsequently computationally analyzed for transcription factor binding motifs using hypergeometric optimization of motif enrichment (HOMER) analysis. There was a significant enrichment in MEF2A and MEF2C binding sites in active enhancers, consistent with the established role of MEF2 in pathological signaling pathways in the heart [95]. Curiously, while this study described a distinction between MEF2A and MEF2C DNA binding sites it is presently unknown whether MEF2 isoforms actually bind to different MEF2 consensus sequences. Taken together, these genome-wide studies reinforce the notion that the MEF2 family of transcription factors are key constituents in enhancers modulated in the progression of cardiac disease.

Finally, a mathematical modeling approach was used to identify putative cardiac enhancers in the human genome. A combination of Gibbs sampling and linear regression was utilized to identify significantly enriched transcription factor binding sites from a large data set of previously validated enhancers active in cardiac development and differentiation [96]. Among the known and de novo transcription factor binding motifs, MEF2 and SRF binding sites obtained the maximum positive weight scores. In a complementary set of computational experiments, co-occurring binding motifs, including MEF2, and other sequence features were used to predict thousands of putative cardiac enhancers in noncoding regions of the human genome. This study supports extensive experimental data that MEF2 is a major player in cardiac gene regulatory networks.

## 4. Direct Cardiomyocyte Reprogramming/Transdifferentiation

As a terminally differentiated tissue, the mammalian heart lacks the ability to replace lost cardiomyocytes. Promising strategies in regenerative medicine have focused on ways to repair diseased hearts that have significant myocyte loss by either stimulating proliferation of pre-existing cardiomyocytes or directly differentiating cardiomyocytes from non-muscle cells in the heart. The latter approach, known as direct reprogramming or transdifferentiation, has been performed successfully in vitro and in mice to generate functional cardiomyocytes from fibroblasts.

Seminal studies on identifying a master transcriptional regulator that drives cardiomyocyte differentiation from a non-muscle source resulted in the finding that a small collection of transcription factors was capable of directly inducing the differentiation of cardiomyocytes from fibroblasts. Overexpressing a cocktail consisting of only three core cardiac transcription factors: GATA4 (G), MEF2C (M), and Tbx5 (T), was sufficient to promote cardiomyocytes from cardiac or skin fibroblasts [97]. Surprisingly, many other combinations of mesodermal and cardiac transcription factors had little to no differentiation inducing effect. Optimization of this procedure showed a more robust transdifferentiation effect with the GMT combination plus the HAND2 transcription factor (GHMT) [98]. It is worth noting that HAND2 was tested in the transdifferentiation assays by Ieda et al. [97] but removal of this factor from the transcription factor cocktail did not significantly reduce the efficiency of direct reprogramming. Nevertheless, these and other studies [99] have found that either GMT or GHMT combination is capable of inducing reprogramming of fibroblasts in the injured (infarcted) heart. Curiously, based on a number of detailed molecular and electrophysiology analyses one study found that the GMT combination could not efficiently direct cardiomyocyte reprogramming from mouse tail tip or cardiac fibroblasts [100]. Along these lines, a recent study revealed that stoichiometry of GATA4, MEF2C, and TBX5, influences the efficiency and quality of induced cardiomyocyte reprogramming [101]. Moreover, overexpression of MEF2C and TBX5 was sufficient to reprogram epigenetically modified cardiac fibroblasts, depleted of polycomb complex gene *Bmi1*, into functional cardiomyocytes [102]. Regardless of the documented differences in transdifferentiation efficiencies, it is clear that MEF2 transcriptional activity is a key component for direct reprogramming in the appropriate context. Because MEF2C was the only MEF2 isoform tested in these experiments it is unknown whether other MEF2 protein isoforms are capable of promoting transdifferentiation. In the future, it would be interesting to determine whether the MEF2 transcriptional requirement in direct reprogramming is restricted to MEF2C or whether other MEF2 family members also display this transdifferentiation ability.

## 5. Concluding Remarks

Here, we have comprehensively reviewed the role of the MEF2 family of transcription factors in the heart of both invertebrate and vertebrate model systems. We have learned a considerable amount about MEF2 function in cardiac development through the genetic dissection of MEF2 orthologs in flies, fish, and mammals, but there is still valuable information that can be extracted regarding their function in cardiomyocytes from these and other model systems with further experimentation. With the availability of genome-wide data describing de novo mutations in human cardiovascular disease, these large data sets could be surveyed for MEF2 mutations and, with current gene editing tools such as CRISPR/Cas9, specific mutations engineered in the MEF2 genes to assess phenotypic effects in vivo. Additional studies will be needed to fully resolve the functionally redundant versus unique roles of the MEF2 family in cardiac morphogenesis. The generation of compound MEF2 mutants particularly with the available conditional MEF2 alleles and isoform knock-in mice would go a long way in addressing this issue.

Given that functional genomics and reprogramming studies on MEF2 have yielded substantial insights into its diverse transcriptional potential, further investigation into the downstream targets of the mammalian MEF2 family in the cardiomyocyte genome and the mechanisms by which these genes are regulated could be achieved through multi-scale genomic approaches. Such global studies could be initially performed in primary myocytes or isolated cardiomyocytes from directed differentiation of pluripotent stem cells and complemented with more technically challenging genome scale analyses in the intact heart. In the future it will also be important to dissect the isoform-specific gene regulatory functions of the MEF2 family at a biochemical and genomic level. Recent evidence suggests that the mammalian MEF2 protein isoforms regulate distinct target genes. While it is firmly established that MEF2 regulates gene expression through interactions with co-factors, which do not appear to discriminate among family members in vitro, the possibility of isoform-specific co-regulators has not been fully realized. A combination of MEF2 isoform binding in the context of chromatin and proteomic analysis of the MEF2 isoform-specific cardiomyocyte interactome with sufficient stringency to minimize common interactions among family members, may be approaches that begin to uncover the molecular mechanisms of transcriptional specificity within this core cardiac transcription factor. This information will help us understand not only the nodal role of MEF2 in cardiac gene regulation but also better define the intricate gene regulatory network in the mammalian heart, with the ultimate goal of developing precision therapies for the treatment of a spectrum of cardiovascular diseases.

## Figures and Tables

**Table 1:** Summary of cardiac-related phenotypes of MEF2 family transcription factor family misexpression in model systems with one, two, and three-chambered hearts.

Model	MEF2 Isoform	Genetic Manipulation	Phenotype	Ref
*D. melanogaster*	*D-Mef2*	Global Loss-of-Function (LoF)	Cardioblasts are specified, failure to differentiate	[12,13]
*C. elegans*	*CeMef2*	Deletion	No observed phenotype	[15]
*D. rerio*	*mef2aa*	Morpholino knockdown	Normal cardiac morphology	[23]
			Significant decrease in cardiac contractility	
		Morpholino knockdown (Bmp2)	Significant loss of Mef2a expression	[24]
			Decrease in cardiac contractility rescued by Mef2a overexpression	
	*mef2ca*	Morpholino knockdown	Delayed cardiomyocyte marker expression	[22]
			Cardiac development delayed, normal heart	
		Global LoF	Delayed cardiomyocyte marker expression	[22]
			Cardiac development delayed, normal heart	
	*mef2cb*	Morpholino knockdown	Secondary heart field defects	[20,22]
			Loss of cardiomyocytes in arterial poles	
			Chamber shortening	
		Global LoF	No observed phenotype	[22]
	*mef2ca/cb*	Global LoF	Pericardial edema	[22]
			Impaired cardiomyocyte differentation	
			Impaired heart tube formation	
	*All mef2*	Morpholino knockdown	Loss of cardiomyocyte differentiation	[22]
			Lack of α-MHC expression	
*X. laevis*	*mef2a*	mRNA microinjection	Precocious expression of α-MHC	[30]
			Enlarged heart	
	*mef2c*	Morpholino knockdown	Cardiac looping defect	[29]
			Chamber expansion defect	
			Mef2D overexpression rescues phenotype	
	*mef2d*	Morpholino knockdown	Cardiac looping defect	[29]
			Chamber expansion defect	
			Mef2C overexpression fails to rescue phenotype	

**Table 2:** Summary of cardiac-related phenotypes of MEF2 family transcription factor family misexpression in mammalian model systems (four chambered hearts).

Model	MEF2 Isoform	Genetic Manipulation	Phenotype	Ref
*M. musculus*	*Mef2a*	Global Loss-of-Function (LoF)	80% **Perinatal lethality**	[39,42]
			Severe myofibrillary defects	
			Dysregulated costamere gene expression	
			20% **Survival to adulthood**	[39,42]
			Mitochondrial deficiency	
			Conduction abnormalities	
		Cardiac-specific LoF(FAK)	Signficiant downregulation of *Mef2a*	[45]
			Embryonic lethality, defective chamber wall maturation, reduced cardiomyocyte proliferation	
		Cardiac overexpression	Dilated cardiomyopathy	[40,82,83]
	*Mef2c*	Global LoF	Embryonic lethality	[48,49,50]
			Defective cardiac looping morphogenesis	
			Vascular malformations	
		CM-specific LoF @~E10.5	Viable embryo	[51]
			Normal cardiac development	
		Double LoF (Mef2c/Nkx2.5)	Development of a single chamber heart	[52]
			Expression of atrial and secondary heart field markers	
		SHF LoF	Outflow tract defects	[53]
			Overriding aorta and double outlet right ventricle	
		Cardiac overexpression	Dilated cardiomyopathy	[83]
	*Mef2d*	Global LoF	Viable , normal cardiac structure and function	[62]
			Attenuated hypertrophy and fibrosis in response to stress	
		Cardiac overexpression	Atrial enlargement	[62]
			Extensive fibrosis	
	*All Mef2*	Dominant Negative	In vitro: impaired cardiomycyte differentiation	[79]
			In vivo: failure to form a heart (severe)	
			thin-walled myocardium (mild)	
		Transgenic Dominant Negative	Attenuated myocardial growth	[81]
			No observable phenotype	[82]
*R. norvegicus*	*Mef2a*	shRNA depletion	Costamere dysregulation	[42]
			Cell Death	
	*Mef2d*	shRNA depletion	Cell cycle re-entry	[63]
			Apoptosis	
